# Involvement of Plasmalogens in Post-Natal Retinal Vascular Development

**DOI:** 10.1371/journal.pone.0101076

**Published:** 2014-06-25

**Authors:** Sarah Saab, Bénédicte Buteau, Laurent Leclère, Alain M. Bron, Catherine P. Creuzot-Garcher, Lionel Bretillon, Niyazi Acar

**Affiliations:** 1 CNRS, UMR6265 Centre des Sciences du Goût et de l’Alimentation, Dijon, France; 2 INRA, UMR1324 Centre des Sciences du Goût et de l’Alimentation, Dijon, France; 3 Université de Bourgogne, UMR Centre des Sciences du Goût et de l’Alimentation, Dijon, France; 4 Department of Ophthalmology, University Hospital, Dijon, France; The University of Melbourne, Australia

## Abstract

**Objective:**

Proper development of retinal blood vessels is essential to ensure sufficient oxygen and nutrient supplies to the retina. It was shown that polyunsaturated fatty acids (PUFAs) could modulate factors involved in tissue vascularization. A congenital deficiency in ether-phospholipids, also termed “plasmalogens”, was shown to lead to abnormal ocular vascularization. Because plasmalogens are considered to be reservoirs of PUFAs, we wished to improve our understanding of the mechanisms by which plasmalogens regulate retinal vascular development and whether the release of PUFAs by calcium-independent phospholipase A2 (iPLA2) could be involved.

**Methods and Results:**

By characterizing the cellular and molecular steps of retinal vascular development in a mouse model of plasmalogen deficiency, we demonstrated that plasmalogens modulate angiogenic processes during the early phases of retinal vascularization. They influence glial activity and primary astrocyte template formation, endothelial cell proliferation and retinal vessel outgrowth, and impact the expression of the genes involved in angiogenesis in the retina. These early defects led to a disorganized and dysfunctional retinal vascular network at adult age. By comparing these data to those obtained on a mouse model of retinal iPLA2 inhibition, we suggest that these processes may be mediated by PUFAs released from plasmalogens and further signalling through the angiopoietin/tie pathways.

**Conclusions:**

These data suggest that plasmalogens play a crucial role in retinal vascularization processes.

## Introduction

Vascular growth occurs through two complementary mechanisms: vasculogenesis and angiogenesis [1]. Vasculogenesis corresponds to the initial vascular tree formation by differentiation of vascular endothelial lineage precursor cells, whereas fine endothelial cell extensions arise by sprouting from pre-existing vessels during angiogenesis. In primates, the retina vascularizes as laminar networks that sequentially radiate peripherally from the optic nerve head [2]. Whereas all vascular laminae emerge post-natally in several mammal species, the innermost plexus arises at gestational age in humans, while the deeper vascular laminae are formed at around 24 weeks of gestation and continue developing after birth [2]. During retinal vascular development, nutrients are supplied to the anterior eye by hyaloid vessels extending from the optic disc. In the growing eye, the development of the retinal vasculature coincides with hyaloid vasculature regression [3]. The hyaloid vascular system fully regresses before birth in humans and during the first post-natal weeks in mice.

While developing, the retinal vasculature associates several cell types. The first stage of retinal vascular development is the formation of the astrocytic bed [4]. The migration of astrocytes from the optic nerve to the retinal periphery is closely followed by the formation of the primary vascular network by endothelial cells [5]. Distinct microglial populations also migrate across the retina prior to or concomitantly with the vessels [6]. Finally, the stabilization of immature vessels by pericytes appears either simultaneously with blood flow or soon thereafter [7].

Dysregulation of retinal vascularization is a common feature of several blinding diseases including retinopathy of prematurity (ROP), diabetic retinopathy (DR) and age-related macular degeneration (AMD) [8]–[10]. In DR and ROP, neovascular events occur at the level of retinal vessels and result in complications such as vitreous haemorrhages, torsional retinal detachment and subsequent blindness [9], [10], whereas choroidal neovascularization is responsible for vision loss in patients with neovascular AMD [8]. Since vascular development is tightly regulated by complex molecular interactions stimulating or inhibiting vasculogenesis and angiogenesis, the pathophysiological mechanisms involved in these diseases may include an imbalance between pro- and anti-angiogenic compounds.

Within the different factors influencing vascular growth, polyunsaturated fatty acids (PUFAs) are drawing interest. In the retina, the major PUFAs are found primarily in neuronal and vascular cell membrane phospholipids from which they are released by phospholipases A2 (PLA2) [11]. Recent discoveries include *in* *vitro* data showing that PUFAs or their metabolites control the expression of pro-angiogenic growth factors in vascular cells [12]–[14], *in* *vivo* animal studies where dietary omega-3 PUFAs reduced pathological angiogenesis [15], and a large-scale human studies associating a higher dietary intake in omega-3 PUFAs with a slower progression of neovascular AMD [16]–[18].

**Figure 1:**
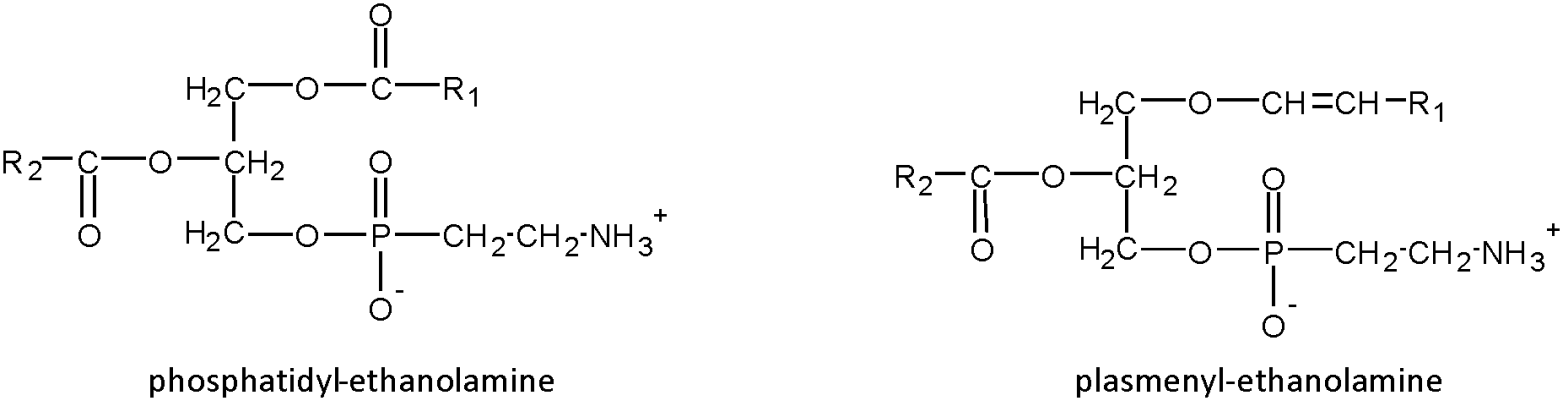
Structure of conventional phospholipids and plasmalogens. Conventional phospholipids such as phosphatidyl-ethanolamine contain ester bonds to link R1 and R2 acyl-moieties at the *sn-*1 and *sn-*2 positions of glycerol, respectively. Ethanolamine-plasmalogens (also termed plasmenyl-ethanolamine) are characterized by the presence of a vinyl-ether bond at the *sn-*1 position of the glycerol backbone to link alkenyl-moieties and an ester bond at the *sn-*2 position to link acyl-residues.

Not only PUFAs but also their phospholipid origin may be important in the control of vascular growth. Indeed, phospholipids in cell membranes can have different sub-types: conventional phospholipids on which fatty acids are connected through ester linkages or specific phospholipids termed “plasmalogens” where a vinyl–ether bond replaces an ester linkage (**Figure 1**). We have shown that plasmalogens accounts for 13% of retinal phospholipids and about 30% of retinal ethanolamine phospholipids [19]. Given that plasmalogens are also considered to be reservoirs of PUFAs in membranes, they are suspected of having signalling functions by releasing these PUFAs through a specific calcium-independent PLA2 (iPLA2) [20]. This hypothesis is reinforced by studies showing higher iPLA2 activities in various pathologic conditions involving plasmalogen metabolism [21], [22]. The importance of plasmalogens in retinal vascular development was previously suggested in a mouse model of plasmalogen deficiency (DAPAT^−/−^ mice). DAPAT−/− mice are characterized by a targeted disruption of the gene encoding for dihydroxyacetone-phosphate acyltransferase, the first enzyme of plasmalogen biosynthesis. The main phenotypic characteristics of DAPAT−/− mice consisted of reduced levels of docosahexaenoic acid (DHA) in neural tissue and complex and severe developmental defects of the central nervous system, the testis and the eye. The ophthalmologic examination of DAPAT^−/−^ mice elicited abnormalities including persistent hyaloid vessels [23]. This feature is likely to be associated with other defects in retinal vasculature that have not been adequately investigated so far.

We therefore characterized blood vessel development in the eye of DAPAT^−/−^ mice and compared the morphologic defects to those of a mouse model of retinal iPLA2 inhibition we developed previously [24]. By correlating these observations with the gene expression of important angiogenic factors, we collected data elucidating the processes by which plasmalogens, and subsequently iPLA2, influence retinal vascular growth. These data demonstrated a temporary delay in retinal vessel outgrowth associated with glial activation, then a secondary sub-numerous and defective development of retinal capillaries, and subsequent scarring processes involving glial and inflammatory cells.

## Materials and Methods

### Animals

Experiments were conducted in accordance with the Association for Research in Vision and Ophthalmology statements and with French legislation (authorization number 21CAE086 for N.A. and animal quarters agreement number A21231010 EA), after approval by the local ethics committees (#105 *Comité d’Ethique de l’Expérimentation Animale Grand Campus Dijon*). C57BL/6 mice (12 weeks old, 20–25 g) were obtained from Elevage Janvier (Le-Genest-Saint-Isle, France). DAPAT heterozygous (DAPAT^+/−^) mutants were kindly provided by Prof. W.W. Just (Heidelberg, Germany). The animals were housed in animal quarters under controlled temperature (22±1°C) and light conditions (12-h light, 12-h dark cycle). Animals were fed *ad libitum* with standard laboratory chow and water.

DAPAT^+/−^ mice were backcrossed with C57Bl/6 mice to provide DAPAT wild-type (DAPAT^+/+^), and DAPAT^+/−^ animals. DAPAT^+/−^ couples were further crossed to generate DAPAT^+/+^, DAPAT^+/−^ and DAPAT knock-out (DAPAT^−/−^) mice. The genotype of heterozygous, DAPAT^+^/^+^ and DAPAT−/− mice was determined according to Rodemer et al. (2003) with slight modifications [23]. The genotype was determined using nested PCR of genomic tail DNA using the primers neomycin-forward (CGCATCGCCTTCTATCGCCTTCTTG, Eurofins MWG Operon, Ebersberg, Germany), exon7-forward (CGATACCTACTTTGTCCCAATTAGC, Eurofins) and exon7-reverse (GCTGGTCTCAAACAGCTACGTAGCTGA, Eurofins). DNA extraction was performed using the Archive Pure DNA Cell/Tissue kit (5 Prime GmbH, Catalog no 2300820; Gaithersburg, MD). Pure genomic DNA was dosed on the nanodrop spectrophotometer (ND 1000, Labtec; Palaiseau, France). For the amplification step, 2 ng of genomic DNA, 100 pmol of each primer in reaction buffer and 2.5 U of Taq polymerase (BiotaqTM DNA Polymerase, BIO-21040, Bioline, Paris, France) were used in a total volume of 25 µl. After 2 min of denaturation at 95°C, PCR was performed on a C1000TM Thermel Cycler (Biorad Laboratories, Hercules, CA) through 35 cycles at 94°C for 30 s and 57°C for 1 min, followed by a final extension step at 72°C for 1 min. This resulted in a 650-bp product for the wild-type gene and an 860-bp product for the neomycin-recombinant DAPAT gene. PCR products were analysed using agarose gel electrophoresis and gels were visualized on Gel doc 2000 (Biorad).

### Inhibition of retinal iPLA2 in DAPAT^+/+^ mice

Retinal iPLA2 was inhibited *in* *vivo* according to previously described procedures [24]. Briefly, retinal iPLA2 was inhibited by 45–55% in DAPAT^+/+^ pups from birth to post-natal days 7 (PN7), 14 (PN14) or 21 (PN21), by repeated intraperitoneal administration of a bromoenol lactone solution (BEL, B1552, Sigma-Aldrich, Saint-Quentin-Fallavier, France) at a concentration of 6 µg/g of body weight in DMSO/saline (1∶10, v:v). Controls were injected with vehicle (DMSO/saline (1∶10, v:v)) only.

### Immunostainings on flat-mounted whole retinas

The pups were euthanized by CO_2_ exposure, the eyeballs were isolated and fixed in 4% paraformaldehyde. The corneas were incised, the lenses taken out, and four radial cuts were made on the eyecups. Vitreous bodies were removed with forceps, and retinas were delicately isolated and flattened on microscope slides.

Flat-mounted retinas were stained to visualize endothelial cells, astrocytes, pericytes and extracellular matrix, as described elsewhere [25]. Briefly, retinas (n = 7–8) were incubated in a blocking solution (1% BSA, PBS-Tween (0.5%), pH 6.8), washed in PBLec solution (PBS, 0.1 mM CaCl2, 0.1 mM MgCl_2_, pH 6.8) and then incubated in a biotin conjugated-isolectin-B4 solution (ILB4, Lectin from *Bandeiraea simplicifolia* BS-I, L2140, Sigma-Aldrich) for endothelial cell labelling. ILB4 solution was prepared at a concentration of 1 µg/µl in 0.9% saline and further diluted in PBLec solution (1∶50, v:v) prior to assay. The retinas were then washed in PBS and incubated in a 0.01 µg/µl streptavidin-Alexa 568 solution (pH 7.2) (S11226, Invitrogen, Saint Aubin, France) prepared in the blocking solution which in turn was prediluted to 50% in PBS. For astrocyte labelling, polyclonal rabbit anti-mouse glial fibrillary acidic protein antibody was used (GFAP, 1∶200, Neomarkers, RB-087-A0,-A1, Illkirch, France). For pericyte and extracellular matrix labelling, polyclonal rabbit anti-chondroitin sulfate proteoglycan antibody (NG-2, 1∶200 Chemicon, Molsheim, France) and polyclonal goat anti-fibronectin antibody (1∶100, Santa Cruz Biotechnology, Le Perray-en-Yvelines, France) were used, respectively. Secondary antibodies were Alexa 488-labelled goat anti-rabbit (1∶200, Invitrogen A11008) and Alexa 488-labelled donkey anti-goat (1∶200, Invitrogen A11055). Following the labelling steps, the retinas were rinsed in PBS and coverslipped using a fluorescence-mounting medium. Controls for these experiments consisted of removing primary antibodies. Fluorescence microphotographs were taken using a Nikon microscope (model Eclipse E600, Nikon, Champigny-sur-Marne, France) and a Nikon digital camera (model OXm 1200C, Nikon) equipped with the Nikon Nis-element BR V2.2 software. Confocal fluorescent micrographs were obtained using a Leica scanning laser confocal microscope SP2 AOBS (Leica Microsystemes SAS, Nanterre, France) and processed with Leica LCSlite.

### Capillary morphometry on flat-mounted whole retinas

Retinal vascular network outgrowth was evaluated on ILB4-stained flat-mounted retinas by calculating the ratio of outgrowth distance to retinal radius. ILB4-positive angiogenic sprouts were counted manually by two operators on unlabelled pictures of flat-mounted retinas displayed at the same magnification and taken from DAPAT^+/+^, DAPAT^−/−^ mice, and mice with retinal iPLA2 inhibition (n = 10–12 per group).

To evaluate retinal capillary density, adult DAPAT^+/+^ and DAPAT^−/−^ mice were deeply sedated using a ketamine (70 µg/g of body weight) and xylazine (14 µg/g of body weight) solution and then perfused with a FITC-dextran solution (molecular weight 2,000,000, Sigma Aldrich) through the left ventricle. Flat-mounted retinas were visualized under a Leica confocal laser-scanning microscope SP2, AOBS (Leica Microsystemes SAS), and processed with Leica LCSlite. After drawing a circle centred on the optic nerve head and covering the entire retina, two additional concentric circles with a radius equivalent to one and two-thirds of the first circle were drawn to delimit retinal central, mid-peripheric and far-peripheric areas. The number of capillaries was determined by drawing an additional concentric circle in the middle of each zone and counting the number of capillaries crossing this ring over 360°. The evaluations were made manually by two operators on unlabelled pictures displayed at the same magnification.

### Microscope characteristics

For the Leica confocal laser-scanning microscope SP2, Acousto Optical Beam Splitters (AOBS), we used the following objectives: 10 HC PL APO CS 10×0.4 dry, 20 HC PL APO CS 20×0.7 dry and 40 HC PL APO CS 40×1.25 oil. The light source excitation for the Alexa 488 and the Alexa 568 fluorochromes was an Argon laser set at 488 (emission spectrum, 507–547 nm) and a helium/xenon laser set at 543 (emission spectrum, 574–657 nm, 600–701 nm or 608–686 nm), respectively. The Nikon microscope (Eclipse E600, Champigny-sur-Marne, France) was equipped with a Nikon digital camera (Nikon OXm 1200C equipped with the Nikon Nis-element BR software V2.2). We used the 4×0.1 dry, 10×0.3 dry and 20×0.5 dry objectives. A mercury lamp was used as a light source, with an excitation spectrum of 450–490 nm with DM 505 and BA 520 for Alexa 488 fluorochrome and 510–560 nm with DM 575 and BA 590 for Alexa 568 fluorochrome.

### Gene expression analyses in retinas

#### Extraction of total RNAs

After the animals were deeply sedated with an intraperitoneal injection of a ketamine (70 µg/g of body weight) and xylazine (14 µg/g of body weight) solution, retinas were isolated from the eyeballs and pooled for one animal (n = 4–6 per group). Total RNAs were isolated using the Ambion RNAqueous kit (AM1912, Life Technologies) according to the manufacturer’s instructions. The quantity and the quality of RNAs were evaluated on a nanodrop spectrophotometer (ND1000, Thermo Fisher, Illkirch, France).

#### cDNAs synthesis and quantitative real-time polymerase chain reaction (PCR)

For quantitative real-time PCR, RNAs were reverse-transcripted to cDNA using the Invitrogen SuperScript VILO™ Master Mix (No. 11755, Life Technologies). The expression of genes coding for known pro- and anti-angiogenic factors [25], inflammatory factors, the glial cell marker and endogenous control genes (**Table 1**) was quantified using 5 ng of total cDNAs in 10 µl of 1X TaqMan Fast Advanced Master Mix (No. 4444557, Applied Biosystems, Life Technologies). Quantitative real time-PCR was performed on TaqMan Array 96-well FAST Plates (Applied Biosystems, Life Technologies), using the StepOnePlus Real-Time PCR System equipped with the StepOne software V2.2.2 (Applied Biosystems, Life Technologies). Data were analysed using DataAssist software V3.0 (Applied Biosystems, Life Technologies). Genes coding for glucuronidase-beta, beta-2-microglobulin and hypoxanthine guanine phosphoribosyl-transferease-1 were used as endogenous controls for normalization and relative quantification (RQ) with the *Cycle Threshold* (C_T_)-method.

**Table 1:** Symbol, name, assay ID, and GenBank reference of assayed genes.

GeneSymbol	Gene name;celera annotation	Assay ID	GenBankreference
***angpt1***	angiopoietin 1; mCG113640	Mm00456503_m1	NM_009640.3
***angpt2***	angiopoietin 2; mCG1200	Mm00545822_m1	NM_007426.3
***efnb2***	ephrin B2; mCG17314	Mm01215897_m1	NM_010111.5
***ephb4***	Eph receptor B4; mCG6855	Mm01201157_m1	NM_001159571.1;NM_010144.6
***fgf2***	fibroblast growth factor 2;mCG12672	Mm00433287_m1	NM_008006.2
***flt1/vegfr1***	FMS-like tyrosine kinase 1;mCG121647	Mm00438980_m1	NM_010228.3
***fn1***	fibronectin 1; mCG121782	Mm01256744_m1	NM_010233.1
***fzd4***	frizzled homolog 4 (Drosophila);mCG15148	Mm00433382_m1	NM_008055.4
***gfap***	glial fibrillary acidic protein;mCG7451	Mm01253033_m1	NM_001131020.1;NM_010277.3
***itgaV***	integrin alpha V; mCG7872	Mm00434486_m1	NM_008402.2
***itgb3***	integrin beta 3; mCG11220	Mm00443980_m1	NM_016780.2
***kdr/vegfr2***	kinase insert domain protein receptor;smCG7209	Mm01222421_m1	NM_010612.2
***pdgfb***	platelet-derived growth factor;B polypeptide; mCG11519	Mm00440677_m1	NM_011057.3
***pdgfrb***	platelet-derived growth factorreceptor; beta polypeptide;mCG6019	Mm00435546_m1	NM_001146268.1;NM_008809.2
***pla2g6***	phospholipase A2; group VI;mCG128876	Mm00479527_m1	NM_001199024.1;NM_001199025.1; NM_016915.4
***tek/tie1***	endothelial-specific receptortyrosine kinase; mCG122568	Mm00443243_m1	NM_013690.2
***thbs1***	thrombospondin 1; mCG14570	Mm00449032_g1	NM_011580.3
***tie1***	tyrosine kinase withimmunoglobulin-likeand EGF-like domains 1; mCG120003	Mm00441786_m1	NM_011587.2
***vegfa***	vascular endothelial growth factor A;mCG2676	Mm01281449_m1	NM_001110267.1; NM_009505.4;NM_001110266.1; NM_001025250.3
**Control genes**			
***b2m***	beta 2 microglobulin	Mm00437762_m1	NM_009735.3
***gusB***	glucuronidase beta	Mm00446953_m1	NM_010368
***hprt***	hypoxanthine guaninephosphoribosyl transferase 1	Mm01545399_m1	NM_013556

### SLO imaging of hyaloid vasculature

The regression of hyaloid vasculature was followed between PN21 and PN27 using a Heidelberg Retina Angiograph confocal scanning laser ophthalmoscope (cSLO, Heidelberg Engineering, Dossenheim, Germany) as previously described [25], [26]. Prior to cSLO angiography, animals were anaesthetized with a ketamine (70 µg/g of body weight) and xylazine (14 µg/g of body weight) solution. After the pupils were dilated with tropicamide (Mydriaticum, Thea Laboratories, Clermont-Ferrand, France), a custom-made contact lens was placed on the cornea using methylcellulose solution (Methocel 2%, OmniVision, Puchheim, Germany). Fluorescein angiography was performed using the Argon laser of the HRA (488 nm; barrier filter: 500 nm), and after a sub-cutaneous injection of a fluorescein-Na solution in 0.9% NaCl at a dose of 75 mg/kg of body weight.

### Electroretinography

The electroretinography equipment consisted of a Ganzfeld bowl, a DC amplifier and a computer-based control and recording unit (RETI port/scan 21, Stasche & Finger GmbH, Roland Consult, Brandenburg, Germany). The electroretinograms (ERGs) (n = 6 per group) were obtained according to previously published procedures [27].

### Statistical analysis

The results are expressed as the mean ± standard deviation (SD) or standard error of the mean (SEM). Statistical analyses were performed using the Statistical Analysis System (SAS Institute, Cary, NC). The non-parametric Kruskal-Wallis test was used between the different groups. Statistical significance was accepted at *P*<0.05. For the statistical analysise of gene expression, the DataAssist software V3.0 (Applied Biosystems, Life Technologies) was used to compare groups two by two through a two-tailed Student's t-test on the DeltaC_T_ values.

## Results

### Temporary delayed retinal vascular outgrowth associated with modifications in the expression of genes involved in angiogenesis

**Figure 2:**
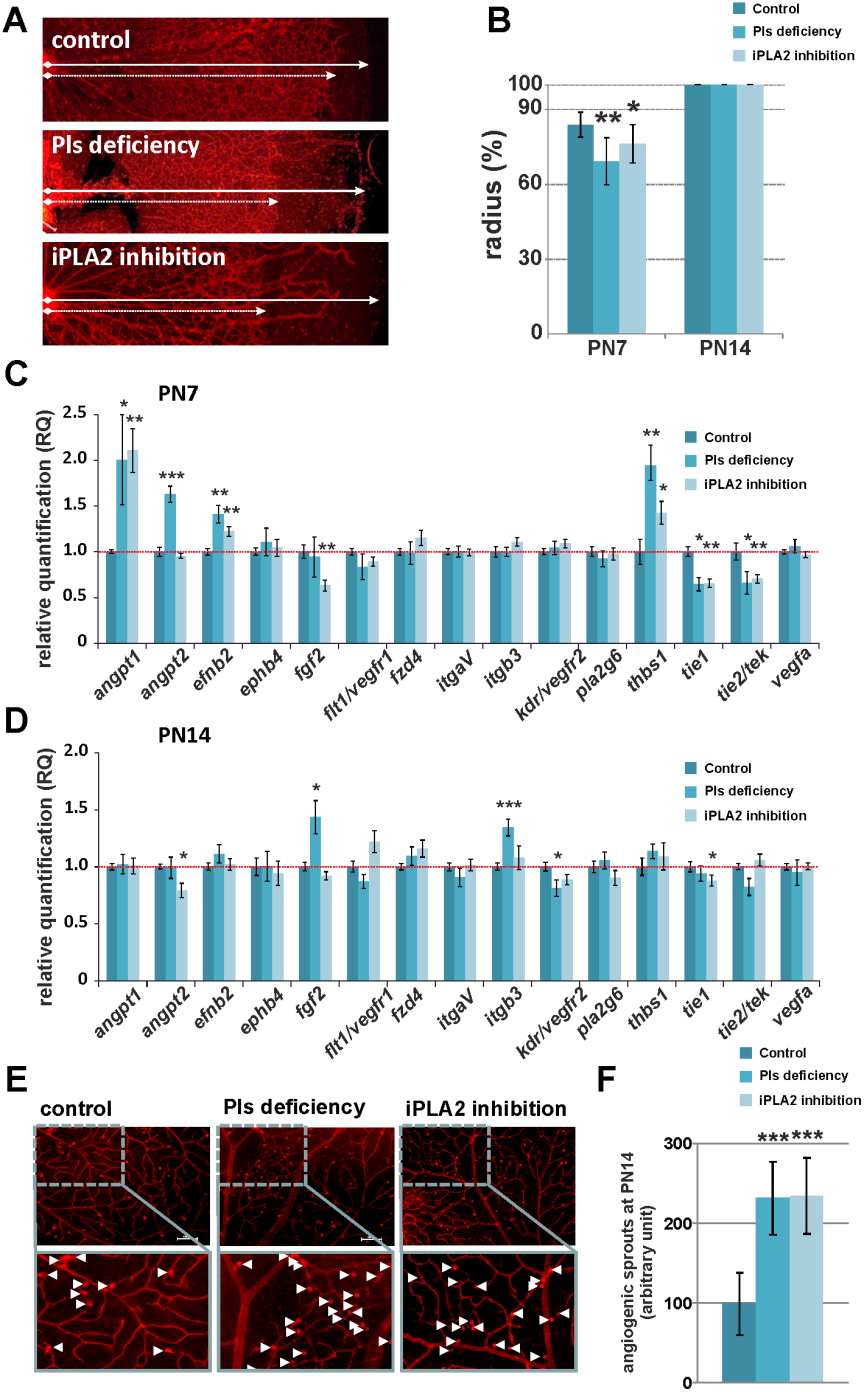
Temporarily delayed outgrowth and increased retinal angiogenic activity in Pls-deficient and iPLA2-inhibited animals. **A.** Representative fluorescence microscopy pictures of isolectin-B4-labelled (ILB4) endothelial cells on retinal whole mounts of control, Pls-deficient and iPLA2-treated mice at PN7. Dashed and solid arrows indicate the outgrowth distance and the retinal radius, respectively. **B.** Quantitative analysis of retinal outgrowth represented by the ratio (%) of outgrowth distance (l) to retinal radius length (L) from the optic nerve to the retinal periphery (R = l/L×100). A delay in retinal vascular outgrowth was observed in Pls-deficient mice and iPLA2-inhibited mice at PN7 (n = 6–18 per group) but not at PN14 (n = 6–10 per group). *: statistically significant difference when compared to control group (Kruskal-Wallis test, *P*<0.05); **: statistically significant difference when compared to control group (Kruskal-Wallis test, *P*<0.01). **C. and D.** Transcriptional analysis of angiogenic factors in retinas of control, Pls-deficient and iPLA2-inhibited mice (n = 6–8 per group) at PN7 (**C.**) and PN14 (**D.**). The relative expression of angiogenic genes was normalized to *gusb*, *hprt* and *b2m* genes and compared to control levels (set as 1). Pls-deficient and iPLA2-treated mice displayed fluctuations in the expression of genes encoding for pro- and anti-angiogenic proteins that were related to vascular phenotype. *: Statistically significant difference when compared to control group (Student’s t-test, *P*<0.05); **: statistically significant difference when compared to control group (Student’s t-test, *P*<0.01); ***: statistically significant difference when compared to control group (Student’s t-test, *P*<0.001). **E.** Representative pictures of ILB4-labelled retinal wholemounts showing angiogenic sprouts (arrowheads) in control, Pls-deficient and iPLA2-inhibited mice at PN14. **F.** Quantitative evaluation of angiogenic sprouts in retinas of control, Pls-deficient, and iPLA2-inhibited mice at PN14 (n = 10–12 per group). The number of angiogenic sprouts was significantly increased in mice deficient in Pls and in animals displaying a chemical inhibition of iPLA2 when compared to control mice (set as 100), suggesting greater sprouting activity. ***: Statistically significant difference when compared to control group (Kruskal-Wallis test, *P*<0.001).

Morphometric evaluation of ILB4-stained retinal vasculature revealed a significantly reduced outgrowth of retinal capillaries at PN7 in mice with plasmalogen deficiency (69% of radius) and iPLA2 inhibition (76% of radius) when compared to control mice (84% of radius) (**Figures 2A and 2B**). These observations were correlated to a significant up-regulation of the anti-angiogenic gene *thrombosporin-1* (*thbs1*) in both plasmalogen-deficient mice and retinal iPLA2-inhibited mice. The delay in vascular outgrowth was also associated with down-regulation of endothelial transcripts coding for the angiopoietin1/2 receptor tie2/tek, and the orphan receptor tie1 in both plasmalogen-deficient and iPLA2-inhibited mice. Among the pro-angiogenic genes, only *angiopoietin-1* (*angpt1*) and *ephrin-B2* (*efnb2*) genes were significantly over-expressed in both mice models (**Figure 2C**). Angpt1 protein is a critical actor involved in vessel maturation since it mediates migration, adhesion and survival of endothelial cells, whereas Efnb2 protein is involved in angio-proliferative retinopathy [28], [29]. We also observed a significant over-expression of the *angiopoietin-2* (*angpt2*) gene in plasmalogen-deficient mice. Angpt2 protein is known to disrupt the connections between the endothelium and perivascular cells, thus promoting cell death and vascular regression [28].

The dysregulation in the expression of these pro- and anti-angiogenic genes was completely abolished in plasmalogen-deficient mice at PN14 (**Figure 2D**). Only *beta-3 integrin* (*itgb3*) and *fibroblast growth factor 2* (*fgf2*) genes − which are known to promote angiogenesis and wound healing − were up-regulated in plasmalogen deficiency conditions. These modifications in gene expression at PN14 were associated to complete centrifuge development of retinal vessels in both mice models (**Figure 2B**). To check whether the recovery of retinal vascular outgrowth was due to greater angiogenic activity, we further quantified the ILB4-stained angiogenic sprouts at PN14 (**Figures 2E and 2F**). The number of angiogenic sprouts was significantly higher in retinas of plasmalogen-deficient mice (350 AU±21.72) and mice with retinal iPLA2 inhibition (290 AU±12.2) compared to controls (247 AU±14.47), suggesting ongoing active angiogenesis processes. This suggests the existence of a secondary increase of pro-angiogenic activity in these animal models that was consistent with the up-regulation of the *itgb3* and *fgf2* genes in retinas of plasmalogen-deficient mice.

### Defects in fully grown retinal vessels at adult age

**Figure 3:**
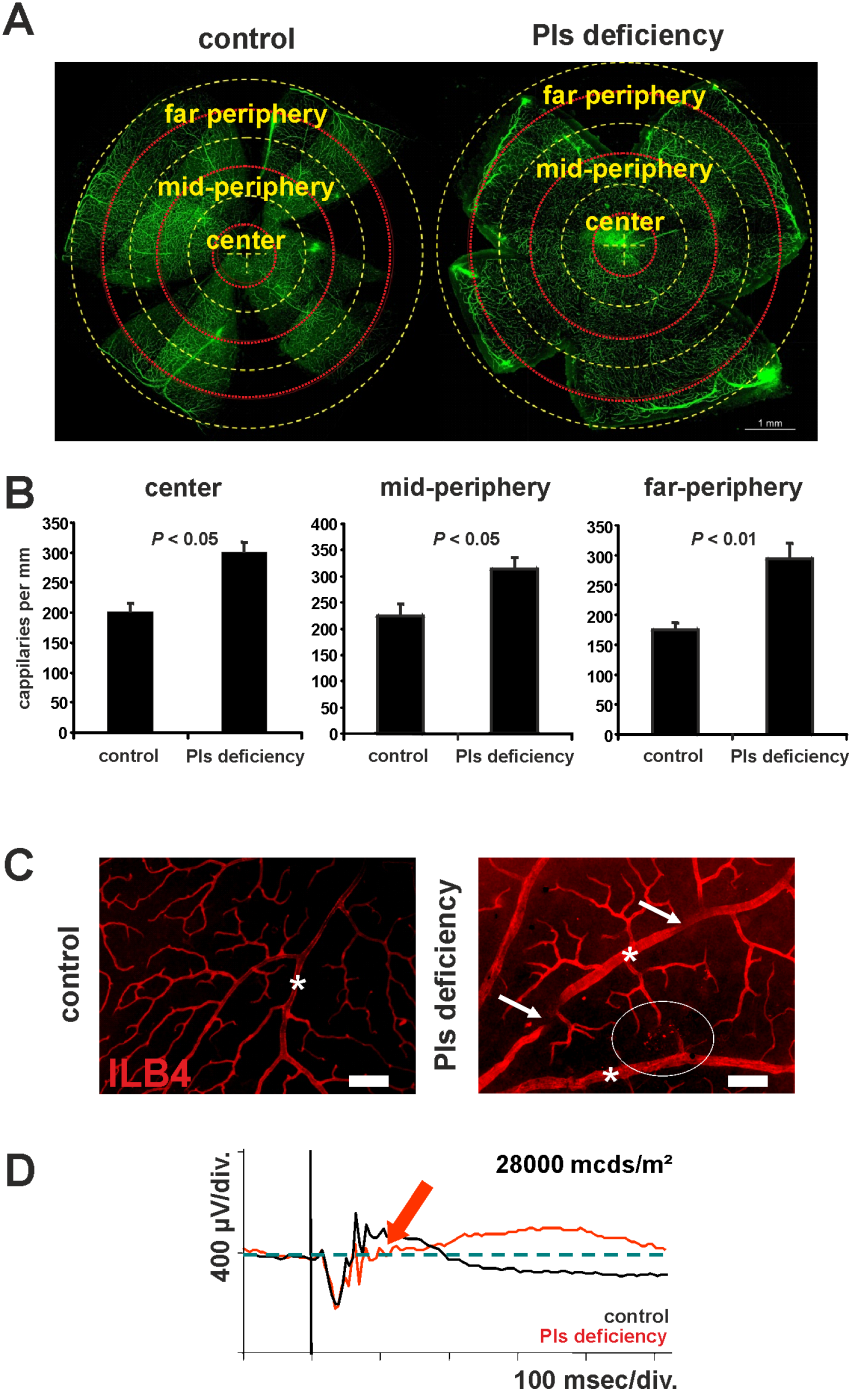
Defects in fully developed retinal vasculature of Pls-deficient mice. **A.** Quantification of retinal capillaries on confocal microscopy pictures of whole-mounted and FITC-dextran-perfused retinas from control and Pls-deficient mice. Three concentric circles (yellow dashed circles) centred on the optic nerve head were drawn to delimit central, mid-peripheric and far peripheric areas. The number of capillaries crossing a ring situated in the middle of each area (red dashed circles) was counted over 360°. **B.** Quantitative evaluation of retinal capillaries in adult control and Pls-deficient mice. The capillary density was significantly increased in central, mid-peripheric and far-peripheric areas of the retina of Pls-deficient mice (n = 6/group). **C.** Representative fluorescence microscopy pictures of isolectin-B4-labelled (ILB4) endothelial cells on retinal wholemounts of control and Pls-deficient mice. Retinal vasculature of adult Pls-deficient mice was characterized by tortuous large vessels (arrows), dilated arteries and veins (stars) and vascular lesions (circle). **D.** Representative electroretinographic response of Pls-deficient and control mice. The ERG traces of Pls-deficient mice (red trace) exhibited a specific alteration of the positive b-wave (arrow) that is typical of retinal hypoxia. Scale bar = 75 µm.

To check whether these early metabolic and cellular abnormalities affect the final organization of retinal vessels, we further investigated the retinal vascular phenotype of adult plasmalogen-deficient mice. We quantified retinal capillaries on FITC-stained flat-mounted retinas in adult plasmalogen-deficient mice (aged of more than 6 months) to check whether the increased sprouting activity and the expression of the *itgb3* and *fgf2* genes at post-natal ages would have consequences on capillary density at adulthood. The number of capillaries was strongly increased in plasmalogen-deficient mice over the entire retinal surface (+49% in the central retina, ^+^40% in the mid-periphery and +68% in the far periphery) (**Figures 3A and 3B**). Moreover, microphotographs of ILB4-stained retinal blood vessels taken from adult plasmalogen-deficient mice showed significant vascular defects compared to controls (**Figure 3C**). These included dilated arteries and veins (stars on **Figure 3C**), tortuous large vessels (arrows on **Figure 3C**) and localized punctuated vascular lesions (circle on **Figure 3C**). Based on previous observations [30], [31], these lesions might result from the down-regulation of *tie1*, *tie2* and *kdr* genes that was observed at an earlier age.

The functional analysis of the retina of plasmalogen-deficient mice using electroretinography revealed a specific alteration of the ERG b-wave, thus resulting in a negative ERG waveform (**Figure 3D**). Except for the oscillatory potentials, the ERG traces of plasmalogen-deficient mice closely resembled those obtained from retinas displaying retinal hypoxia [25], [32], thus suggesting that the increased diameter of large vessels may be a secondary consequence of reduced retinal oxygenation, as previously described [33].

### Contribution of extracellular matrix and astrocytes to endothelial cell proliferation during early steps of vascular development

**Figure 4:**
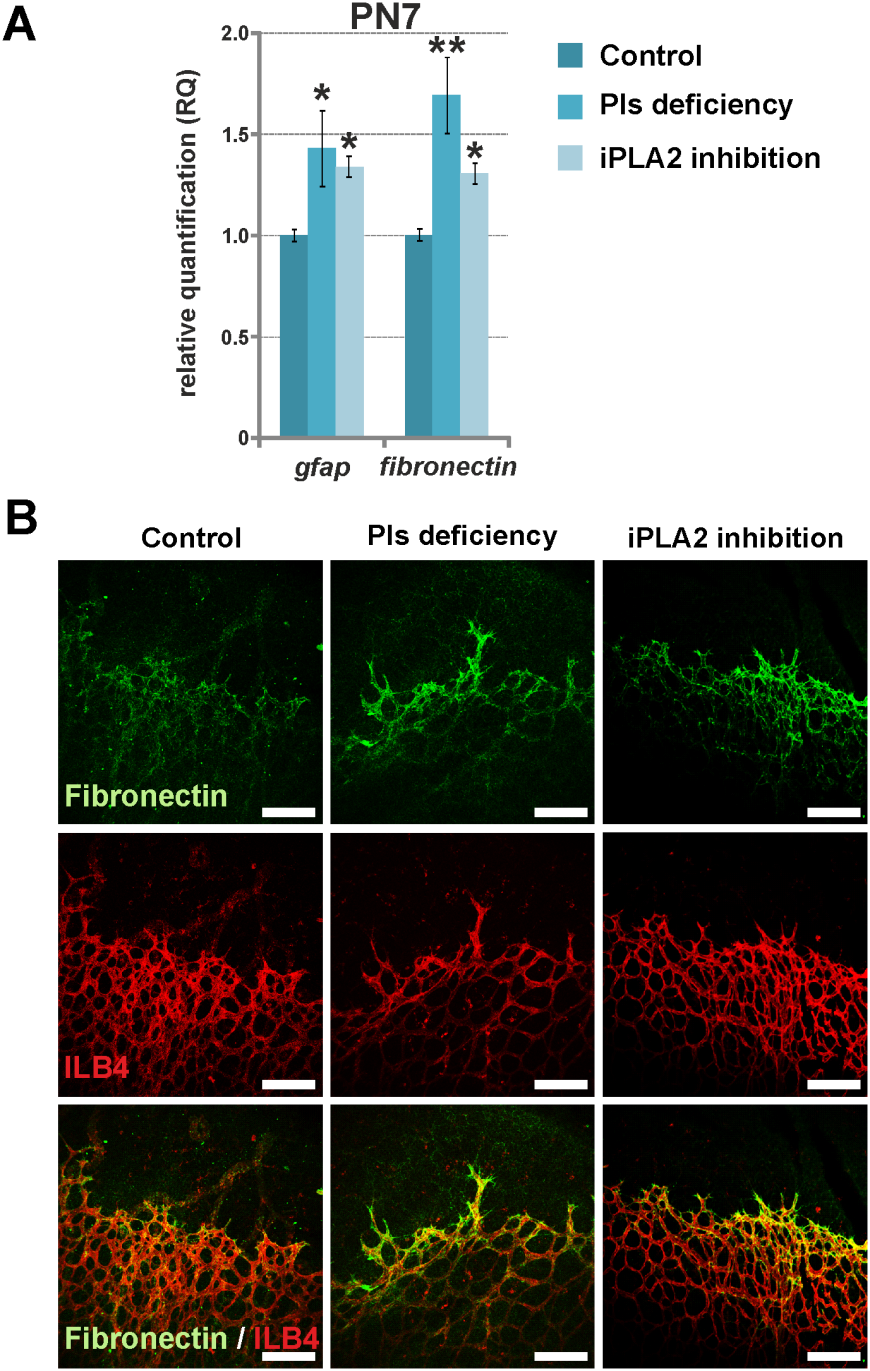
Greater retinal astroglial activity in Pls-deficient and iPLA2-inhibited animals at PN7. **A.** Relative expression of genes encoding for two markers of astroglial activity in control animals (n = 4–5), Pls-deficient mice (n = 5) and iPLA2-treated mice (n = 6) examined by RT-qPCR. The relative expression of *gfap* and *fn1* genes encoding for GFAP and fibronectin, respectively, was normalized to *gusb, hprt* and *b2m* genes and compared to the control level (set at 1). The expression of *gfap* and *fn1* genes was significantly increased in Pls-deficient and iPLA2-treated mice at PN7, suggesting increased astroglial activity. *: Statistically significant difference when compared to control group (Student’s t-test, *P*<0.05); **: statistically significant difference when compared to control group (Student’s t-test, *P*<0.01). **B.** Confocal microscopy of anti-fibronectin (green) and ILB4 (red) labelled retinal whole mounts of control, Pls-deficient and iPLA2-inhibited mice at PN7 (n = 3–6 per group). The secretion of fibronectin protein by retinal astrocytes at the front of vascular outgrowth was more pronounced in Pls-deficient and iPLA2-inhibited animals when compared to controls, confirming greater astroglial activity. Scale bar = 150 µm.

Fibronectin is an extra-cellular matrix protein known to promote endothelial cell proliferation and migration during vascular development [34]. In physiologic conditions, fibronectin is expressed in the zone of vasculogenesis immediately prior to vessel formation, and it was shown to be over-expressed in pathological retinal microvessels [35]. In the animal models used herein, the delayed vascular outgrowth observed at PN7 was associated with up-regulation of *fibronectin* gene expression (**Figure 4A**). At the protein level, fibronectin immuno-reactivity was more intense in the retina of animals with plasmalogen deficiency and iPLA2 inhibition. The protein was particularly expressed around vessels situated at the front of outgrowth, suggesting more active angiogenesis processes in this area (**Figure 4B**). Fibronectin up-regulation was correlated to an increased expression of the *gfap* gene coding for the glial fibrillary acidic protein (GFAP) in the retina (**Figure 4A** **)**. This is consistent with previous studies reporting fibronectin-induced endothelial cell proliferation by *gfap* over-expressing activated astrocytes [34], [36]. These data suggest that astrocytes may be at the origin of the mechanisms promoting angiogenesis in mice with plasmalogen deficiency and iPLA2 inhibition at PN7.

### Influence of astrocyte template on vessel architecture of adult mice

**Figure 5:**
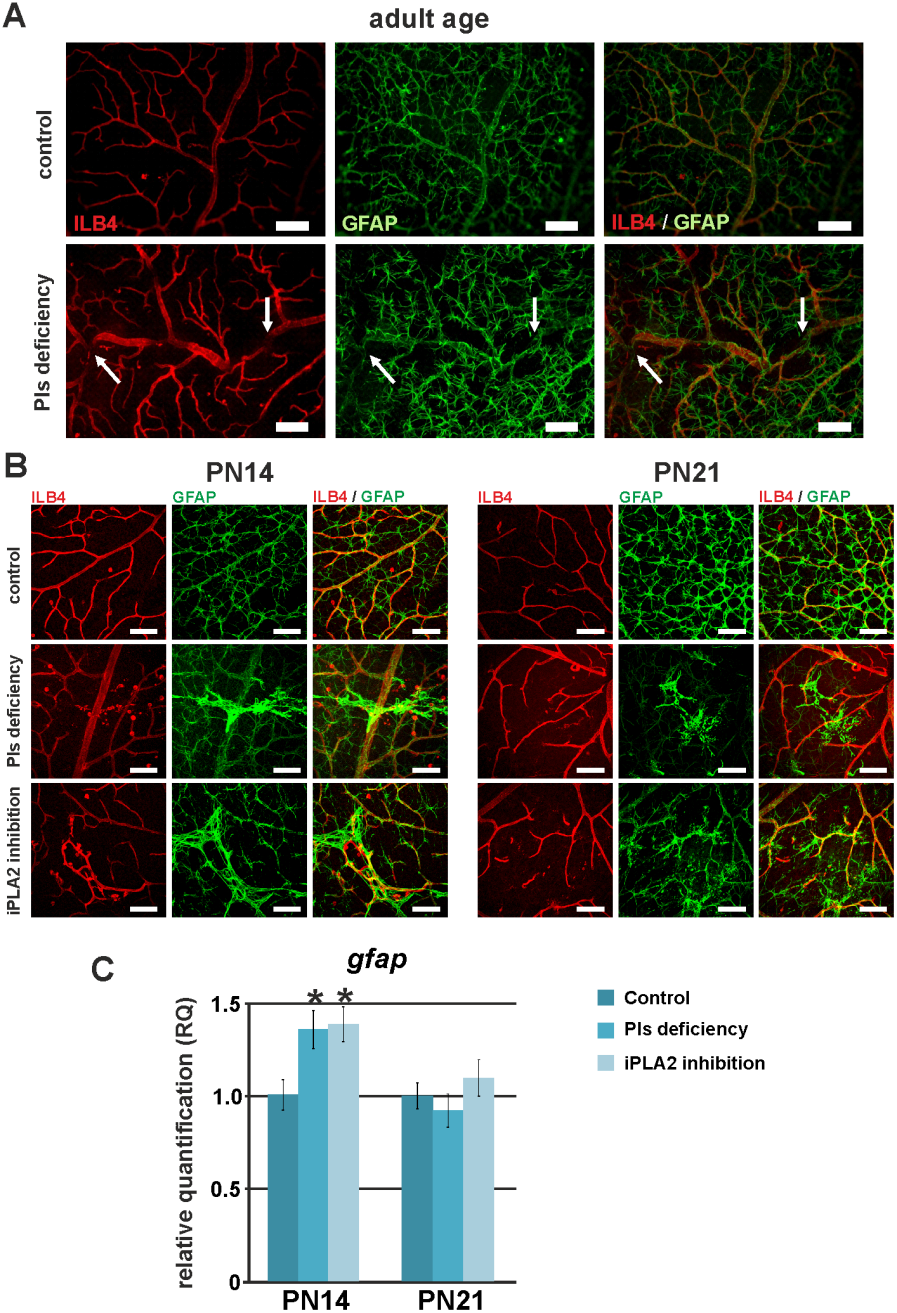
Influence of astrocyte template on vessel architecture of adult mice. **A.** Fluorescence microscopy pictures of anti-GFAP- (green) and isolectin-B4- (ILB4, red) labelled retinal whole mounts of control and Pls-deficient mice and mice at adult age. ILB4-positive cells (endothelial cells) and GFAP-positive cells (astrocytes) were co-localized, suggesting that vessel tortuosity is a secondary consequence of an abnormal arrangement of the astrocytic bed. **B.** Confocal microscopy pictures of anti-GFAP- (green, labelling astrocytes) and isolectin-B4- (ILB4, red, labelling endothelial cells) labelled retinal whole mounts of control, Pls-deficient and iPLA2-inhibited mice at PN14 and PN21. The retinal vasculatures of Pls-deficient and iPLA2-inhibited mice were characterized by vascular lesions that co-localized with activated astrocytes. These activated-astrocyte areas were sharply outlined at PN14, whereas they were less immuno-reactive to GFAP and had a fibrous aspect at PN21. **C.** Relative expression of GFAP, a marker of astroglial activity in control animals, Pls-deficient mice and iPLA2-treated mice examined by RT-qPCR (n = 4–6 per group) at PN14 and PN21. The relative expression of the *gfap* gene was normalized to *gusb*, *hprt* and *b2m* genes and compared to the control level (set at 1). The expression of the *gfap* gene was significantly increased in Pls-deficient and iPLA2-treated mice at PN14, suggesting increased astroglial activity at this age. *: Statistically significant difference when compared to control group (Student’s t-test, *P*<0.05). Scale bar = 75 µm.

As previously reported, a proper astrocyte template is required for retinal vascular development and remodelling [34], [37]. To check whether the vascular tortuosity observed in adult plasmalogen-deficient mice is related to irregularities in astrocyte template formation, we observed ILB4-stained endothelial cells and GFAP-stained astrocytes on retinal flat-mounts from adult DAPAT^−/−^ mice (**Figure 5A**). The co-localization of ILB4- and GFAP-positive cells suggests that endothelial cells have passively followed the defective astrocytic template (arrows on **Figure 5A**). Then, the abnormal spatial positioning of endothelial cells might be the secondary consequence of a defective arrangement of the astrocytic bed.

To better understand the origin of the localized vascular lesions, we further investigated astrocyte template formation and retinal vasculature at PN14 and PN21. In addition to the well-shaped astrocyte bed, we observed several localized, sharply outlined and strongly GFAP-immuno-reactive astrocyte accumulation areas in plasmalogen-deficient mice and in iPLA2-inhibited mice at PN14. The co-localization of ILB4- and GFAP-positive cells showed that these areas corresponded to the sites of vascular lesion development. At PN21, astrocytes appeared to be less accumulated and/or less immuno-reactive than at PN14, to be less sharpened and to have a more fibrous aspect (**Figure 5B**). The areas where astrocytes accumulated were not present at PN7 and seemed to result from secondary scarring mechanisms. Gene expression analysis confirmed up-regulation of retinal GFAP mRNAs at PN14 and a return to control levels at PN21 (**Figure 5C**).

### No impact of plasmalogen deficiency and iPLA2 inhibition on pericyte recruitment

**Figure 6:**
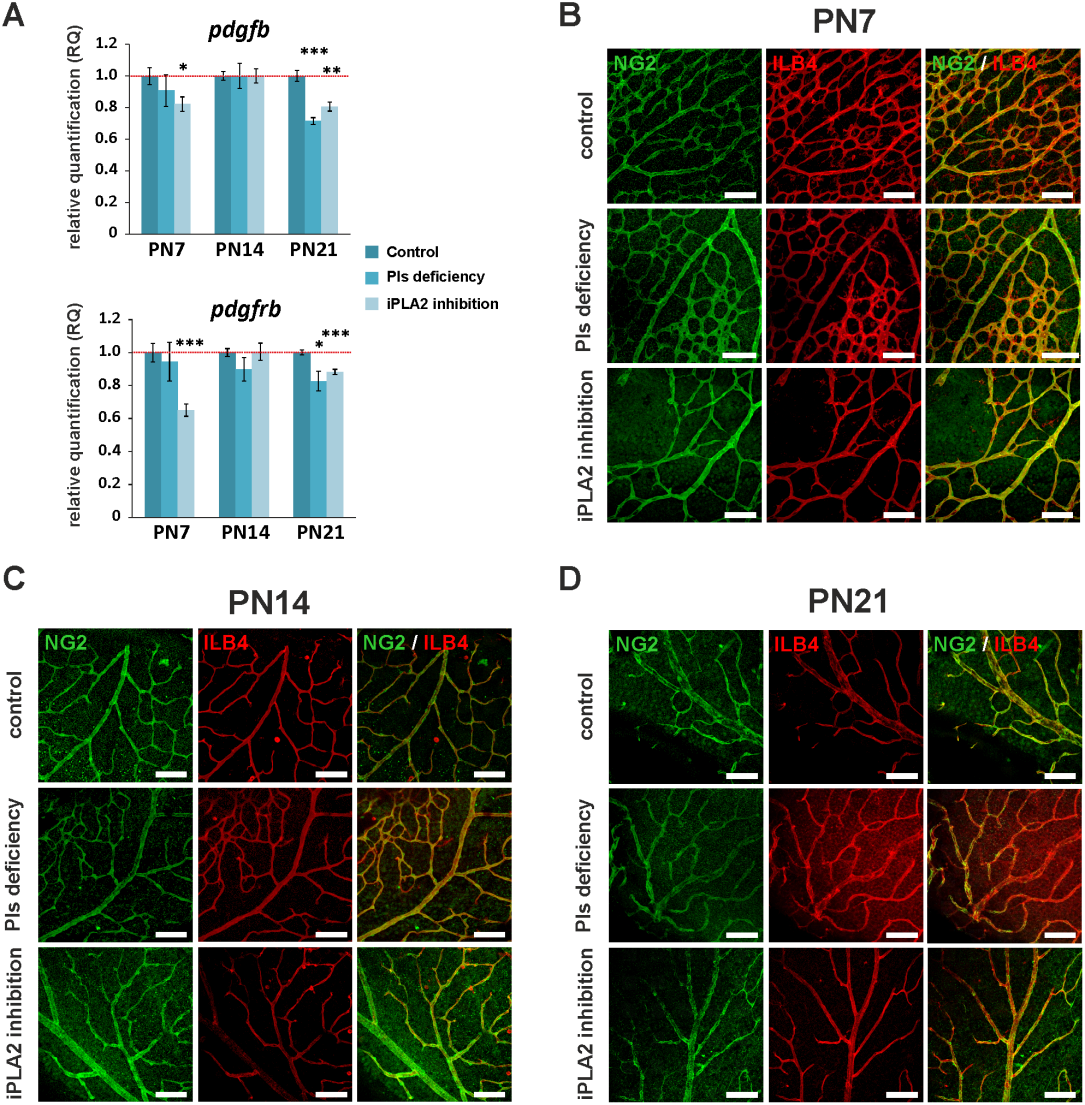
Pericyte recruitment and vessel stabilization in retinas of control, Pls-deficient and iPLA2-inhibited mice. **A.** Relative expression of *PDGF* and *PDGFR* in control animals, Pls-deficient mice and iPLA2-treated mice examined by RT-qPCR (n = 4–6 per group) at PN7, PN14 and PN21. The relative expression of the genes was normalized to *gusb, hprt* and *b2m* genes and compared to the control level (set at 1). The expression of *pdgfb* and *pdgfrb* genes was significantly reduced in mice with iPLA2 inhibition at PN7 and in Pls-deficient and iPLA2-inhibited mice at PN21. *: Statistically significant difference when compared to control group (Student’s t-test, *P*<0.05); **: statistically significant difference when compared to control group (Student’s t-test, *P*<0.01); ***: statistically significant difference when compared to control group (Student’s t-test, *P*<0.001). **B., C. and D.** Confocal microscopy pictures of anti-NG2- (green, labelling pericytes) and isolectin-B4- (ILB4, red, labelling endothelial cells) labelled retinal whole mounts of control and Pls-deficient mice and mice at PN7 (**B.**), PN14 (**C.**) and PN21 (**D.**). No abnormality was observed in retinas of Pls-deficient and iPLA2-inhibited mice, suggesting normal vessel stabilization by pericytes. Scale bar = 75 µm.

We speculated that a defective vessel maturation through pericyte recruitment would be involved in vessel dilation and tortuosity, and in the formation of vascular lesions. Because platelet-derived growth factor-beta (PDGF-β) signalling is required for pericyte recruitment and migration [38], we wanted to know whether the expression of *pdgfb* and *pdgfrb* genes is modified in the retinas of our mice model. Slight but significant down-regulation of *pdgfb* and *pdgfrb* genes was observed at PN21 (**Figures 6A**). However, immuno-stainings of retinal pericytes with anti-NG2 antibody did not reveal any impact of plasmalogen deficiency or iPLA2 inhibition on pericyte recruitment and positioning next to vessels, at any stage of development (**Figure 6B**).

### Delayed regression of hyaloid arteries in mice with retinal iPLA2 inhibition

**Figure 7:**
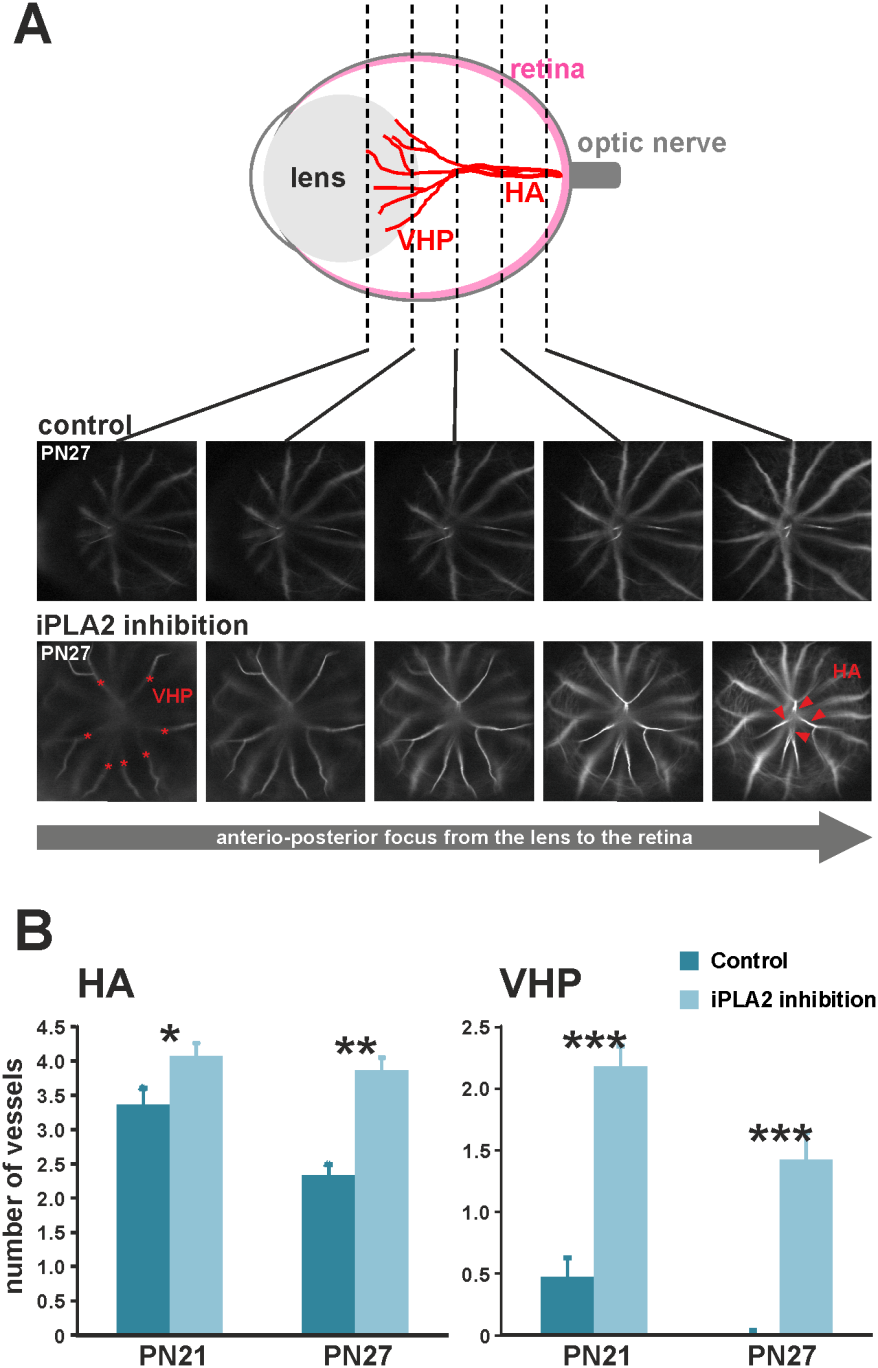
Defects in hyaloid vasculature regression in mice with iPLA2 inhibition. A. Quantification of hyaloid arteries (HA) and *vasa hyaloidea propria* (VHP) vessels on depth-scan images from confocal cSLO angiography. VHP vessels (stars) were visualized and quantified at the level of the posterior lens, whereas HAs (arrowheads) were counted in the posterior eye in control and iPLA2-inhibited animals (n = 11 per group). **B.** Quantitative evaluation of hyaloid arteries (HA) and *vasa hyaloidea propria* (VHP) vessels in control and iPLA2-inhibited mice at PN21 and PN27. The numbers of HAs and VHPs were significantly higher in iPLA2-inhibited mice at PN21 and PN27 when compared to controls, thus confirming that the control of hyaloid vessel regression by Pls involves the iPLA2 enzyme. *: Statistically significant difference when compared to control group (Kruskal-Wallis test, *P*<0.05); **: statistically significant difference when compared to control group (Kruskal-Wallis test, *P*<0.01); ***: statistically significant difference when compared to control group (Kruskal-Wallis test, *P*<0.001).

A persistent hyaloid vasculature was previously reported in plasmalogen-deficient mice [23]. As evidence of the implication of plasmalogens and iPLA2 in retinal vascular development, the regression of hyaloid vasculature in animals with iPLA2 inhibition was observed (**Figure 7**). We performed fluorescein angiography on iPLA2-inhibited mice at PN21 and PN27 to visualize and quantify the functional hyaloid vessels, namely hyaloid arteries (HAs) arising from the optic nerve and entering the vitreous and *vasa hyaloidea propria* (VHP), which are small vessels branched to HAs and radiating in the vitreous (**Figure 7A**) [39]. With the confocal module of the angiograph, we identified and quantified individual VHPs at the level of the lens and HAs in the posterior eye. As expected, HAs were present in greater quantities in iPLA2-inhibited mice at PN21 (mean ± SEM, 4.07±0.19 and 3.36±0.24 in treated and control animals, respectively; **Figure 7B**) and were persistent in iPLA2-inhibited mice, while they regressed in controls (−5% (non-significant) and −31% (*P*<0.05) between PN21 and PN27 in treated and control animals, respectively). The effect of iPLA2 inhibition was even more striking for VHPs, whose number was greater in iPLA2-inhibited animals (mean ± SEM, 2.15±0.16 and 0.45±0.15 in treated and control animals, respectively) at PN21 and still persistent at PN27, whereas they were fully regressed in control animals. This evidence could also explain the delay in retinal vessel outgrowth at earlier stages, as was previously hypothesized [25].

## Discussion

Based on a preliminary description of the ocular phenotype of a mouse model of plasmalogen deficiency [23] and to the well-known implication of PUFAs in angiogenesis [15], [40], [41], we hypothesized that plasmalogens may participate in the control of retinal vascular development through PUFA release by a phospholipase belonging to iPLA2 family. To test this hypothesis, we characterized the steps of retinal vascular development in plasmalogen-deficient mice and in a previously developed mouse model of retinal iPLA2 inhibition [24].

We found that plasmalogens were involved in the control of the early steps of retinal vascular development. During these phases, endothelial cell proliferation results in the formation of the primary vascular bed on an astrocyte template. As shown by the present data, plasmalogen deficiency results in delayed vascular outgrowth and in an imbalance in the retinal expression of pro- and anti-angiogenic genes. These anti-angiogenic conditions were associated with high macroglial activity and the resulting over-production of fibronectin. Astrocytes seem to be the principal actor, since fibronectin production was reported to be linked to astrocyte activity. Fibronectin is known to promote endothelial cell proliferation and vessel formation in the retina [34], [42] and was found to be over-expressed in retinal capillaries of patients with proliferative DR, a disease displaying an abnormal proliferation of retinal vessels [35]. Nevertheless, further studies are needed to decipher any involvement of Müller cells, which are also known to promote endothelial cell proliferation and to be immuno-reactive to anti-GFAP antibodies. Although the production of fibronectin was enhanced, it was not sufficient, at least at PN7, to balance the lack of development of retinal vessels observed at this age. One hypothesis would be that this greater astroglial activity is a secondary reaction of the retina to delayed vascular development. This process might have been mediated by transient hypoxia, since some data demonstrated an increased secretion of fibronectin in hypoxic astrocytes in the brain [43] and the retina [36].

Whereas the expression of VEGF was not affected by plasmalogen deficiency or iPLA2 inhibition, the only up-regulated pro-angiogenic factors at PN7 belong to the angiopoietin family. Angiopoietins are involved in vessel formation through the control of endothelial cell migration, adhesion and survival. Angiopoietins have been found to be ligands of tie receptors [28]. The present results indicate that the retinal expression of endothelial transcripts, such as tie2 and tie1 [44], does not follow that of angiopoietins at PN7. On the contrary, the mRNAs of tie receptors decreased, whereas those of angiopoietins increased. These findings may indicate either a lower relative number of endothelial cells in the retina or an altered activity of the angiopoietin/tie pathway, which may contribute to the delayed formation of the endothelial network. Since angiopoietin production was previously shown to be negatively regulated by omega-3 PUFAs [13], one may assume that the over-expression of angiopoietins is a consequence of the lack of plasmalogens and the subsequent PUFA signalization. Further analyses of the kinases involved in these pathways would be helpful to confirm this hypothesis and to elucidate the molecular mechanisms involved. Furthermore, and as suggested by Hoffman et al., the over-expression of the *angpt1* gene may also have influenced retinal vessel maturation through mechanisms not involving pericytes [45]. Although pericytes are known to participate in vessel stabilization and maturation, these cells may not be involved in the formation of mature vessels in animal models of ROP. These data are concordant with our results, as plasmalogen-deficient mice exhibit features resembling to those observed in ROP, namely vascular growth retardation, proliferation of retinal capillaries, and dilated and tortuous arteries and veins.

Several genetic and phenotypic changes occurred between PN7 and PN14. The retinal vascular outgrowth was boosted in plasmalogen-deficient and iPLA2-inhibited animals, as confirmed by the increased sprouting activity at PN14 and the higher number of retinal capillaries at adulthood. This higher angiogenic activity was confirmed at the molecular level by the return to control levels of pro-angiogenic gene expression, whereas these were down-regulated at PN7. Only the *itgb3* and *fgf2* genes remained up-regulated in plasmalogen-deficient mice at PN14. The *itgb3* gene encodes for beta3-integrin protein, which is considered as a marker of angiogenesis [46]. Although the pro-angiogenic properties of beta-3-integin are still under debate in cancer research [47], studies have shown that its activation enhances tumour angiogenesis and metastatic growth in the brain [48]. Another particularity of integrins is their ability to interact with a number of pro-angiogenic factors such as EGF, PDGF-beta and IGF [47]. Such interactions may be important for the activation of integrins as well as for the regulation of kinase activity of these growth factors [49]. Even if this type of interaction has not yet been demonstrated with FGF-2, several reports suggest that beta-3 integrin and FGF-2 are involved in common metabolic pathways in vascular or ocular cells [50]–[52]. Whereas FGF-2 itself is known to be an important actor in wound healing [53], it was demonstrated that beta-3 integrin is also required for wound angiogenesis [54]. Taken together, these data suggest that FGF-2 and beta-3 integrin may have promoted the scarring processes observed in plasmalogen-deficient and iPLA2-inhibited mice at PN14. These wound areas were characterized by strongly activated astrocytes on which angiogenic processes take place. These scarring processes were very transitory and incomplete at PN21, thus leading to punctuated vascular lesions at later ages.

One of the vascular abnormalities observed in fully developed retinas of plasmalogen-deficient and iPLA2-inhibited mice consisted of defects in large vessels. We observed an up-regulation of genes encoding for angpt1, angpt2 and Efnb2 proteins, which are known to be over-expressed in the retinas of animal models of oxygen-induced retinopathy and in the vitreous of patients with ROP. Oxygen exposure is also known to promote vessel regression and up-regulate *Efnb2* gene expression [29], [45], [55]. The *angpt2* gene was shown to be over-expressed in patients with highly vascular-active ROP [55] and was only up-regulated in plasmalogen-deficient mice, suggesting a more severe phenotype in mice with plasmalogen deficiency than in those with retinal iPLA2 inhibition. The tortuosity of large vessels can result from either poor positioning of astrocytes when forming the astroglial template or a secondary modification of the extracellular matrix that subsequently modified the vessel shape. Given that omega-3 PUFAs were shown to influence the tissue expression of several proteins of the extracellular matrix [56], [57], this second hypothesis would be attractive.

As final evidence of the involvement of an iPLA2-dependent control of vascular development by plasmalogens, we have shown another phenotypic similarity between our mouse models, namely the persistence of hyaloid vasculature. Based on previous observations, one might assume that the defective regression of hyaloid vasculature may also be related to alterations in astrocyte metabolism, and particularly their ability to produce βA3/A1-crystallin protein, further work being needed to document this hypothesis [37].

There are a several limitations to acknowledge in this work. First, and to better characterize the influence of plasmalogens on the early steps of retinal vascular development, it would have been useful to increase the number of time points for a complete time course of retinal development. Second, and since this study is mainly based on data obtained from mRNA analyses, the tissue levels of the different proteins could have been evaluated by western blotting or ELISA. Third, the inhibition rate of iPLA2 activity in our animal model is only 45%, meaning that about half of the retinal iPLA2 activity is still persistent [24]. Moreover, bromoenol lactone was shown to inhibit plasmalogen-specific isoforms of iPLA2 as well as others that are not specifically related to these phospholipids. Other approaches such as suppression of plasmalogen-specific iPLA2 gene activity by siRNA or by homologous recombination would have been more effective. However, to the best of our knowledge, the nucleotide sequence of the retinal isoform of this plasmalogen-specific enzyme is not known, making these approaches impossible to use.

In summary, plasmalogen deficiency resulted in primary vascular defects that led to a secondary tissue reaction that is insufficient to ensure physiological vascular development. In addition to confirming the need for plasmalogens for normal retinal vascular development, this study provides evidence that helps elucidate the cellular and molecular events involved. Regulation of angiogenesis by plasmalogens can be mediated by the action of iPLA2 and involves the angiopoietin/tie pathway.

## References

[1] Risau W, Flamme I (1995) Vasculogenesis. Annu Rev Cell Dev Biol 11: 73–91. 8689573 10.1146/annurev.cb.11.110195.000445

[2] Gariano RF, Iruela-Arispe ML, Hendrickson AE (1994) Vascular development in primate retina: comparison of laminar plexus formation in monkey and human. Invest Ophthalmol Vis Sci 35: 3442–3455. 8056520

[3] Zhu M, Madigan MC, van Driel D, Maslim J, Billson FA, et al (2000) The human hyaloid system: cell death and vascular regression. Exp Eye Res 70: 767–776. 10843781 10.1006/exer.2000.0844

[4] Kopatz K, Distler C (2000) Astrocyte invasion and vasculogenesis in the developing ferret retina. J Neurocytol 29: 157–172. 11428047 10.1023/a:1026594721760

[5] Dorrell MI, Aguilar E, Friedlander M (2002) Retinal vascular development is mediated by endothelial filopodia, a preexisting astrocytic template and specific R-cadherin adhesion. Invest Ophthalmol Vis Sci 43: 3500–3510. 12407162

[6] Diaz-Araya CM, Provis JM, Penfold PL, Billson FA (1995) Development of microglial topography in human retina. J Comp Neurol 363: 53–68. 8682937 10.1002/cne.903630106

[7] Fruttiger M (2002) Development of the mouse retinal vasculature: angiogenesis versus vasculogenesis. Invest Ophthalmol Vis Sci 43: 522–527. 11818400

[8] Ambati J, Fowler BJ (2012) Mechanisms of age-related macular degeneration. Neuron 75: 26–39. 22794258 10.1016/j.neuron.2012.06.018 PMC3404137

[9] Antonetti DA, Klein R, Gardner TW (2012) Diabetic retinopathy. N Engl J Med 366: 1227–1239. 22455417 10.1056/NEJMra1005073

[10] Hartnett ME, Penn JS (2012) Mechanisms and management of retinopathy of prematurity. N Engl J Med 367: 2515–2526. 23268666 10.1056/NEJMra1208129 PMC3695731

[11] Fliesler SJ, Anderson RE (1983) Chemistry and metabolism of lipids in the vertebrate retina. Prog Lipid Res 22: 79–131. 6348799 10.1016/0163-7827(83)90004-8

[12] Kermorvant-Duchemin E, Sennlaub F, Sirinyan M, Brault S, Andelfinger G, et al (2005) Trans-arachidonic acids generated during nitrative stress induce a thrombospondin-1-dependent microvascular degeneration. Nat Med 11: 1339–1345. 16311602 10.1038/nm1336 PMC4850227

[13] Szymczak M, Murray M, Petrovic N (2008) Modulation of angiogenesis by omega-3 polyunsaturated fatty acids is mediated by cyclooxygenases. Blood 111: 3514–3521. 18216296 10.1182/blood-2007-08-109934

[14] Zhuang W, Wang G, Li L, Lin G, Deng Z (2012) Omega-3 polyunsaturated Fatty acids reduce vascular endothelial growth factor production and suppress endothelial wound repair. J Cardiovasc Transl Res 6: 287–293. 22993129 10.1007/s12265-012-9409-0

[15] Connor KM, SanGiovanni JP, Lofqvist C, Aderman CM, Chen J, et al (2007) Increased dietary intake of omega-3-polyunsaturated fatty acids reduces pathological retinal angiogenesis. Nat Med 13: 868–873. 17589522 10.1038/nm1591 PMC4491412

[16] Chong EW, Robman LD, Simpson JA, Hodge AM, Aung KZ, et al (2009) Fat consumption and its association with age-related macular degeneration. Arch Ophthalmol 127: 674–680. 19433719 10.1001/archophthalmol.2009.60

[17] Kishan AU, Modjtahedi BS, Martins EN, Modjtahedi SP, Morse LS (2011) Lipids and age-related macular degeneration. Surv Ophthalmol 56: 195–213. 21439604 10.1016/j.survophthal.2010.08.008

[18] Seddon JM, George S, Rosner B (2006) Cigarette smoking, fish consumption, omega-3 fatty acid intake, and associations with age-related macular degeneration: the US Twin Study of Age-Related Macular Degeneration. Arch Ophthalmol 124: 995–1001. 16832023 10.1001/archopht.124.7.995

[19] Acar N, Gregoire S, Andre A, Juaneda P, Joffre C, et al (2007) Plasmalogens in the retina: in situ hybridization of dihydroxyacetone phosphate acyltransferase (DHAP-AT)–the first enzyme involved in their biosynthesis–and comparative study of retinal and retinal pigment epithelial lipid composition. Exp Eye Res 84: 143–151. 17081518 10.1016/j.exer.2006.09.009

[20] Farooqui AA, Farooqui T, Horrocks LA (2008) Roles of plasmalogens in brain. In: New York: Springer Science + Business Media Springer, editor. Metabolism and Functions of Bioactive Ether Lipids in the Brain. LLC: 85–106.

[21] Farooqui AA (2010) Studies on plasmalogen-selective phospholipase A2 in brain. Mol Neurobiol 41: 267–273. 20049656 10.1007/s12035-009-8091-y

[22] Hazen SL, Ford DA, Gross RW (1991) Activation of a membrane-associated phospholipase A2 during rabbit myocardial ischemia which is highly selective for plasmalogen substrate. J Biol Chem 266: 5629–5633. 2005103

[23] Rodemer C, Thai TP, Brugger B, Kaercher T, Werner H, et al (2003) Inactivation of ether lipid biosynthesis causes male infertility, defects in eye development and optic nerve hypoplasia in mice. Hum Mol Genet 12: 1881–1895. 12874108 10.1093/hmg/ddg191

[24] Saab-Aoude S, Bron AM, Creuzot-Garcher CP, Bretillon L, Acar N (2013) A mouse model of in vivo chemical inhibition of retinal calcium-independent phospholipase A2 (iPLA2). Biochimie 95: 903–911. 23266358 10.1016/j.biochi.2012.12.008

[25] Luhmann UF, Meunier D, Shi W, Luttges A, Pfarrer C, et al (2005) Fetal loss in homozygous mutant Norrie disease mice: a new role of Norrin in reproduction. Genesis 42: 253–262. 16035034 10.1002/gene.20141

[26] Seeliger MW, Beck SC, Pereyra-Munoz N, Dangel S, Tsai JY, et al (2005) In vivo confocal imaging of the retina in animal models using scanning laser ophthalmoscopy. Vision Res 45: 3512–3519. 16188288 10.1016/j.visres.2005.08.014

[27] Boudard DL, Acar N, Bretillon L, Hicks D (2011) Retinas of the diurnal rodent Arvicanthis ansorgei are highly resistant to experimentally induced stress and degeneration. Invest Ophthalmol Vis Sci 52: 8686–8700. 21960552 10.1167/iovs.11-8162

[28] Fagiani E, Christofori G (2013) Angiopoietins in angiogenesis. Cancer Lett 328: 18–26. 22922303 10.1016/j.canlet.2012.08.018

[29] Ehlken C, Martin G, Lange C, Gogaki EG, Fiedler U, et al (2011) Therapeutic interference with EphrinB2 signalling inhibits oxygen-induced angioproliferative retinopathy. Acta Ophthalmol 89: 82–90. 19764912 10.1111/j.1755-3768.2009.01609.x

[30] Chen-Konak L, Guetta-Shubin Y, Yahav H, Shay-Salit A, Zilberman M, et al (2003) Transcriptional and post-translation regulation of the Tie1 receptor by fluid shear stress changes in vascular endothelial cells. FASEB J 17: 2121–2123. 14500555 10.1096/fj.02-1151fje

[31] Fujikawa K, Presman E, Isner JM, Varticovski L (1999) Expression of tie1 and tie2 proteins during reendothelialization in balloon-injured rat carotid artery. J Vasc Res 36: 272–281. 10474040 10.1159/000025655

[32] Luu CD, Foulds WS, Kaur C (2012) Electrophysiological findings in a porcine model of selective retinal capillary closure. Invest Ophthalmol Vis Sci 53: 2218–2225. 22427562 10.1167/iovs.11-8490

[33] Rennie D, Morrissey J (1975) Retinal changes in Himalayan climbers. Arch Ophthalmol 93: 395–400. 1131077 10.1001/archopht.1975.01010020409001

[34] Jiang B, Liou GI, Behzadian MA, Caldwell RB (1994) Astrocytes modulate retinal vasculogenesis: effects on fibronectin expression. J Cell Sci 107 (Pt 9): 2499–2508. 10.1242/jcs.107.9.2499 7844167

[35] Roy S, Cagliero E, Lorenzi M (1996) Fibronectin overexpression in retinal microvessels of patients with diabetes. Invest Ophthalmol Vis Sci 37: 258–266. 8603829

[36] Uemura A, Kusuhara S, Wiegand SJ, Yu RT, Nishikawa S (2006) Tlx acts as a proangiogenic switch by regulating extracellular assembly of fibronectin matrices in retinal astrocytes. J Clin Invest 116: 369–377. 16424942 10.1172/JCI25964 PMC1332029

[37] Sinha D, Valapala M, Bhutto I, Patek B, Zhang C, et al (2012) betaA3/A1-crystallin is required for proper astrocyte template formation and vascular remodeling in the retina. Transgenic Res 21: 1033–1042. 22427112 10.1007/s11248-012-9608-0 PMC3773944

[38] Hellstrom M, Kalen M, Lindahl P, Abramsson A, Betsholtz C (1999) Role of PDGF-B and PDGFR-beta in recruitment of vascular smooth muscle cells and pericytes during embryonic blood vessel formation in the mouse. Development 126: 3047–3055. 10375497 10.1242/dev.126.14.3047

[39] Ito M, Yoshioka M (1999) Regression of the hyaloid vessels and pupillary membrane of the mouse. Anat Embryol (Berl) 200: 403–411. 10460477 10.1007/s004290050289

[40] Calviello G, Di Nicuolo F, Gragnoli S, Piccioni E, Serini S, et al (2004) n-3 PUFAs reduce VEGF expression in human colon cancer cells modulating the COX-2/PGE2 induced ERK-1 and -2 and HIF-1alpha induction pathway. Carcinogenesis 25: 2303–2310. 15358633 10.1093/carcin/bgh265

[41] Chong EW, Kreis AJ, Wong TY, Simpson JA, Guymer RH (2008) Dietary omega-3 fatty acid and fish intake in the primary prevention of age-related macular degeneration: a systematic review and meta-analysis. Arch Ophthalmol 126: 826–833. 18541848 10.1001/archopht.126.6.826

[42] Wilson SH, Ljubimov AV, Morla AO, Caballero S, Shaw LC, et al (2003) Fibronectin fragments promote human retinal endothelial cell adhesion and proliferation and ERK activation through alpha5beta1 integrin and PI 3-kinase. Invest Ophthalmol Vis Sci 44: 1704–1715. 12657612 10.1167/iovs.02-0773

[43] Li L, Welser JV, Dore-Duffy P, del Zoppo GJ, Lamanna JC, et al (2010) In the hypoxic central nervous system, endothelial cell proliferation is followed by astrocyte activation, proliferation, and increased expression of the alpha 6 beta 4 integrin and dystroglycan. Glia 58: 1157–1167. 20544851 10.1002/glia.20995 PMC2914614

[44] Sato TN, Tozawa Y, Deutsch U, Wolburg-Buchholz K, Fujiwara Y, et al (1995) Distinct roles of the receptor tyrosine kinases Tie-1 and Tie-2 in blood vessel formation. Nature 376: 70–74. 7596437 10.1038/376070a0

[45] Hoffmann J, Feng Y, vom Hagen F, Hillenbrand A, Lin J, et al (2005) Endothelial survival factors and spatial completion, but not pericyte coverage of retinal capillaries determine vessel plasticity. Faseb J 19: 2035–2036. 16215210 10.1096/fj.04-2109fje

[46] Ruegg C, Alghisi GC (2010) Vascular integrins: therapeutic and imaging targets of tumor angiogenesis. Recent Results Cancer Res 180: 83–101. 20033379 10.1007/978-3-540-78281-0_6

[47] Robinson SD, Hodivala-Dilke KM (2011) The role of beta3-integrins in tumor angiogenesis: context is everything. Curr Opin Cell Biol 23: 630–637. 21565482 10.1016/j.ceb.2011.03.014

[48] Lorger M, Krueger JS, O’Neal M, Staflin K, Felding-Habermann B (2009) Activation of tumor cell integrin alphavbeta3 controls angiogenesis and metastatic growth in the brain. Proc Natl Acad Sci U S A 106: 10666–10671. 19541645 10.1073/pnas.0903035106 PMC2697113

[49] Somanath PR, Ciocea A, Byzova TV (2009) Integrin and growth factor receptor alliance in angiogenesis. Cell Biochem Biophys 53: 53–64. 19048411 10.1007/s12013-008-9040-5 PMC2863046

[50] Hennig T, Mogensen C, Kirsch J, Pohl U, Gloe T (2011) Shear stress induces the release of an endothelial elastase: role in integrin alpha(v) beta(3)-mediated FGF-2 release. J Vasc Res 48: 453–464. 21691119 10.1159/000327009

[51] Meitinger D, Hunt DM, Shih DT, Fox JC, Hunt RC (2001) Vitreous-induced modulation of integrins in retinal pigment epithelial cells: effects of fibroblast growth factor-2. Exp Eye Res 73: 681–692. 11747368 10.1006/exer.2001.1079

[52] Pickering JG, Uniyal S, Ford CM, Chau T, Laurin MA, et al (1997) Fibroblast growth factor-2 potentiates vascular smooth muscle cell migration to platelet-derived growth factor: upregulation of alpha2beta1 integrin and disassembly of actin filaments. Circ Res 80: 627–637. 9130443 10.1161/01.res.80.5.627

[53] Schuschereba ST, Bowman PD, Ferrando RE, Lund DJ, Quong JA, et al (1994) Accelerated healing of laser-injured rabbit retina by basic fibroblast growth factor. Invest Ophthalmol Vis Sci 35: 945–954. 8125757

[54] Mitchell K, Szekeres C, Milano V, Svenson KB, Nilsen-Hamilton M, et al (2009) Alpha3beta1 integrin in epidermis promotes wound angiogenesis and keratinocyte-to-endothelial-cell crosstalk through the induction of MRP3. J Cell Sci 122: 1778–1787. 19435806 10.1242/jcs.040956 PMC2684832

[55] Sato T, Shima C, Kusaka S (2011) Vitreous levels of angiopoietin-1 and angiopoietin-2 in eyes with retinopathy of prematurity. Am J Ophthalmol 151: 353–357 e351. 10.1016/j.ajo.2010.08.037 21168819

[56] Tourtas T, Birke MT, Kruse FE, Welge-Lussen UC, Birke K (2012) Preventive effects of omega-3 and omega-6 Fatty acids on peroxide mediated oxidative stress responses in primary human trabecular meshwork cells. PLoS One 7: e31340. 22319624 10.1371/journal.pone.0031340 PMC3272013

[57] Vardar-Sengul S, Buduneli E, Turkoglu O, Buduneli N, Atilla G, et al (2008) The effects of selective COX-2 inhibitor/celecoxib and omega-3 fatty acid on matrix metalloproteinases, TIMP-1, and laminin-5gamma2-chain immunolocalization in experimental periodontitis. J Periodontol 79: 1934–1941. 18834249 10.1902/jop.2008.080001

